# Comparison of antiplatelet activity of garlic tablets with cardio-protective dose of aspirin in healthy volunteers: a randomized clinical trial

**Published:** 2016

**Authors:** Mojtaba Shafiekhani, Pouya Faridi, Javad Kojuri, Soha Namazi

**Affiliations:** 1 *Department of Pharmacotherapy, school of Pharmacy, Shiraz University of Medical Sciences, Shiraz, Iran*; 2 *Department of phytopharmaceuticals, school of Pharmacy, Shiraz University of Medical Sciences, Shiraz, Iran*; 3 *Department of Cardiology, school of Medicine, Shiraz University of Medical Sciences, Shiraz, Iran*

**Keywords:** *Garlic*, *Aspirin*, *Anti-Platelet*, *Light transmittance aggregometry*

## Abstract

**Objective:**

Some of the adverse effects of aspirin including peptic ulcers, gastrointestinal bleeding and aspirin resistance compelled researchers to find a suitable alternative with fewer adverse effects. In this clinical trial, we aimed to find the effective antiplatelet dose of garlic.

**Materials and Methods:**

This randomized controlled clinical trial (RCT) was conducted on 62 healthy volunteers of 20-50 years old. All volunteers used 80 mg aspirin per day for 1 week and at the end of this time, platelet aggregation (PA) induced by 4 agonists acting in aggregation pathway including adenosinediphosphate (20 μmol/l), epinephrine (20 μmol/l), collagen(0.19 mg/ ml) and arachidonic acid (0.5mg/ ml) was measured by Light Transmittance Aggregometry (LTA) in all participants. After one month washout period, volunteers were randomized into 3 groups and each received 1, 2 or 3 garlic tablets (1250 mg) a day for 1 month. After one month, PA was examined in all groups.

**Results:**

The mean ±SD of the age of all volunteers was 28.60 ± 9.00 years. In addition, 52.00 % of our volunteers were male and 48.00% of them were female. Garlic tablet didnot have significant effect on PA at any dose. However, 30% of volunteers in the group that used 3 garlic tablets/day reported adverse effect (i.e. bleeding). No significant association between sex, age and PA was observed.

**Conclusion:**

In this study, we were unable to determine the effective anti-platelet dose of garlic which that could be equal to that of aspirin anti-platelet activity, as assessed LTA method.

## Introduction

Patients with acute coronary syndrome (ACS) or who undergo percutaneous coronary intervention (PCI) require anti-platelet drugs for inhibition of plaque rupture and then, platelet activation and aggregation which cause thrombosis (Angiolillo, 2009 ▶). Current guidelines suggest anti-platelet therapy in these patients to prevent atherothrombotic events (Kushner et al., 2009 ▶).

ASA (acetyl salicylic acid) and thienopyridine derivatives such as clopidogrel are major components of anti-platelet therapy in current guidelines. Although efficacy of these drugs has been proven for many years, some data show that about 5 to 40 % of coronary patients treated with anti-platelet drugs experienced thrombotic events (Farré et al., 2010 ▶). Aspirin-resistance refers to a proportion of patients who receive aspirin but experience clinical failure and ischemic events. One study estimated the prevalence of aspirin resistance to be 5 to 60 % (Farré et al., 2010 ▶). Also, the phrase "clopidogrel resistance" has recently emerged and the prevalence of this phenomenon has been reported to be 5 to 44 % (Kuliczkowski et al., 2009 ▶).

According to variable response to dual antiplatelet therapy in coronary heart disease and also occurrence of thrombotic events such as myocardial infarction, stroke and cardiovascular death in these patients (Kuliczkowski et al., 2009 ▶, Angiolillo et al., 2009 ▶, Farré et al., 2010 ▶), it is necessary to provide some strategies to overcome this problem or introduce new anti-platelet agents. 

Garlic, with the scientific name *Allium sativum* from Liliaceae family, is perhaps the most commonly accepted plant used as medicine in various cultures for over 7000 years.

In some papers, cholesterol and blood lipids lowering properties and blood pressure reducing and the antioxidant effects of garlic are more emphasized (Lau et al., 1983 ▶, Gupta and Porter, 2001 ▶, Sendl, 1995 ▶). All these activities have made garlic an effective agent for prevention and treatment of cardiovascular diseases. Other recognized properties of garlic are antithrombotic, fibrinolytic and anti-platelet effects (Ernst, 1986 ▶, Schaffer et al., 1996 ▶). The most abundant sulfur containing compound found in garlic is alliin (Schaffer et al., 1996 ▶). When garlic is cut, crushed or chewed, an enzyme [found in garlic cloves] called allinase converts alliin to allicin. Allicin in garlic is the most important and the most effective substance with pharmacological effects (Schaffer et al., 1996 ▶).

Nevertheless, some believe that garlic has another potent compound with anti-platelet effects called ajoene, which is the second product of alliin’s metabolism (Agarwal, 1996 ▶). Afzal et al. showed that ajoen synergistically potentiates the effects of antiplatelet drugs such as forskolin, indomethacin and dipyridamole (Apitz-Castro et al., 1986 ▶, Afzal et al., 2000 ▶).

 Some studies conducted on the anti-platelet effects of garlic mentioned different mechanisms for its anti-platelet activity such as inhibiting cyclooxygenase (Jain et al., 1993 ▶), increasing cAMP level(Wagner et al., 1987 ▶), suppressing calcium metabolism in platelets and increasing nitric oxide (Srivastava, 1993 ▶); but the results of these studies are not definitive about the exact mechanism of garlic. Based on these studies, garlic seems to be a safe and effective anti-platelet agent in primary and secondary prevention of cardiovascular events. 

In our study, anti-platelet effects of garlic were evaluated in a randomized controlled clinical trial (RCT), using four known agonists in the platelet aggregation pathway including adenosine diphosphate (ADP), collagen, epinephrine (EPN), and arachidonic acid (AA). Attempt was also made to find out whether garlic is a proper alternative to aspirin (at its anti-platelet dose (80 mg/day)) or not.

## Materials and Methods

**Patient selection**

This case control, open – labeled, RCT was conducted from June to December 2012. All subjects gave written informed consent. This study was approved by the ethics committee of Shiraz University of Medical Sciences and registered in IRCT (Iranian Registry of Clinical Trial) with registration ID IRCT2012073110453N1.

 From all selected volunteers, 20ml blood samples were taken after 12 hr fasting. Fasting blood glucose (FBS), low density lipoprotein cholesterol (LDL-C), high density lipoprotein cholesterol (HDL-C), triglyceride (TG), total cholesterol, cell blood count (CBC) and basal platelet aggregation with 4 agonists namely, ADP, AA, EPN and collagen for each volunteer were checked. Finally, 62 healthy individuals aged 20-50 years old were enrolled in this RCT. 

Exclusion criteria was applied to individuals had been using aspirin, clopidogrel and nonsteroidal anti-inflammatory drugs (NSAIDs) or any drug affecting platelets function (such as omega-3 and Selective serotonin re-uptake inhibitors (SSRIs)) in the last 2 weeks or throughout the study, or who had cardiovascular diseases, thrombocytopenia (platelet<100×10^3^ platelets/mm^3^), anemia (hemoglobin<10g/dl), abnormal lipid profile(total cholesterol ≥ 200 mg/dl, LDL-C ≥100mg/dl, TG ≥ 200 mg/dl, HDL-C≤ 45 mg/dl for males and ≤ 55 mg/dl for females), diabetes mellitus (FBS ≥ 126 mg/dl confirmed by repeating the test) and also renal failure ( serum creatinine ≥ 2mg/dl for femaleand serum creatinine≥2.5 mg/dl for males)(Longo et al., 2011 ▶). 

**Platelet Aggregation**

Platelet aggregation test was done by Light Transmittance Aggregometry (LTA; HELENA PACKS -4, UK) as follows: 20 ml of blood was obtained from each participant. Blood samples were collected in tubes containing 3.8% Na-citrate. The tubes were then centrifuged for 8 min at 800 rpm to obtain platelet-rich plasma (PRP) and then for 20 min at 4000 rpm to recover platelet-poor plasma (PPP). All tests were done within 2 hr after PRP and PPP preparation. The platelet count in the PRP samples was adjusted to be between 250×10^3^/μl and 300×10^3^/μl with PPP. Platelets were stimulated with final concentration of 0.5mg/ml AA (HELENA Bioscience Europe Sunderland, UK) and 0.19 mg/ ml collagen (HELENA Bioscience Europe Sunderland, UK) and 20μmol/l ADP(HELENA Bioscience Europe Sunderland, UK), and 20μmol/l EPN (HELENA Bioscience Europe Sunderland, UK).

 Platelet aggregation was expressed as the maximal percent change in light transmittance from the baseline value, with PPP as a reference.

**Evaluating platelet aggregation after aspirin intake**

All volunteers received aspirin (Pars Daru Company,Tehran,Iran) 80 mg/day (cardio-protective dose) for one week. After this time, a 20 ml blood sample was obtained and the platelet aggregation was determined by LTA after induction by 20μmol/l AA. Then, for all volunteers a wash out period of 1 month was considered.

**Evaluating platelet aggregation after garlic intake**

Volunteers were randomly divided into 3 groups of 21members by simple randomization method. The first group was given one garlic tablet (garlic 1250mg, odor control, Nature Made, United States) per day, the second group was given two garlic tablets and the third received three garlic tablets (1250mg) per day for one month.

**Figure 1:**
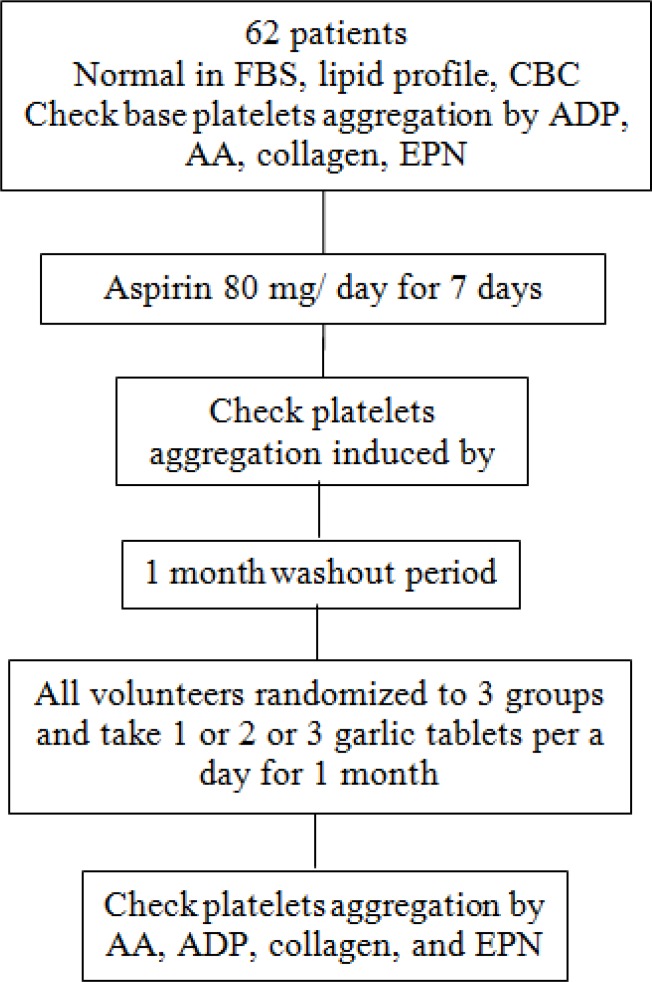
The flowchart of open labeled randomized clinical trial study of the evaluation of anti-platelet effect of different doses of garlic tablets.

A one week supply of garlic tablets were provided to the volunteers weekly and volunteers were also followed by telephone contact, giving and receiving information regarding their garlic use during every one week period and also reporting any observed adverse reaction related to the use of garlic (Figure 1).

During the study, all volunteers were asked not to take any NSAIDs or drugs that affect the platelet aggregation. At the end of 1month, 20 ml blood samples were taken from all volunteers for determination of platelet aggregation. Then, platelets were stimulated with final concentration of 20μmol/l AA and 0.19 mg/ml collagen and 20μmol/l ADP, and 20μmol/l EPN by LTA method.

AA = arachidonic acid, ADP = adenosine phosphate, EPN= epinephrine, CBC= count blood cell, FBS=fasting blood sugar

**Statistical analysis**

The normality of the distribution was checked using Kolmogorov–Smirnov test. Continuous variable was presented as mean±SD. Categorical data was shown as percent and means of continuous variables were compared using student’s t-test. Comparison was made between continuous variables and groups by one way analysis of variance (ANOVA). The effect of sex on platelet aggregation was examined by t-test and the correlation between age and platelet aggregation was assessed by Pearson correlation test.

In this study, a p-value≤0.05 was considered significant. All analyses were performed using the SPPS 18.0 (SPSS, Inc., IBM Company, Chicago, Illinois) statistical software.

## Results

The mean±SD of the age of all volunteers was 28.60±9.00 years. Fifty two percent of our volunteers were male and 48.00% of them were female. The demographic and clinical information of the volunteers are presented in Table 1. According to Table 1 there were no significant differences among the three groups regarding the demographic and basal laboratory data (p>0.05).

**Table 1:** Demographic data and laboratory tests results of healthy volunteers consumed different doses of garlic tablets. (N = 62

	** Group1 ** **( 1 tablet/day) **	**Group2** **(2tablets/day)**	**Group3** **(3tablets/day)**	**Total **	**P** ^1^ **(value)**
**Sex. No(%)**					
**Male ** **Female **	9 10	12 10	12 9	33(54) 29(46)	0.886 0.886
**Age, year, mean±SD**	26.21±8.00	27.88±9.20	31.74±9.80	28.61±9.00	0.067
**FBS, mg/dl, mean±SD**	91.89 ±7.72	90.77±5.60	91.90±5.94	91.49±6.42	0.822
**BUN, mg/dl, mean±SD**	14.21±3.30	14.18±2.97	15.95±3.66	14.77±3.31	0.424
**Serum creatinine, mg/dl, mean±SD**	1.06±0.11	1.06±0.13	1.10±0.18	1.077±0.14	0.446
**Total cholesterol mg/dl, mean±SD**	181.36±64.30	170.04±53.18	198.25±59.12	182.81±59.20	0.551
**Triglyceride mg/dl, mean±SD**	114.78±50.18	119.95±51.22	116.15±58.35	117.09±53.25	0.681
** LDL- C mg/dl, mean±SD**	105.63±30.21	99.27±27.90	101.94±22.68	102.13±26.93	0.3477
**HDL-C mg/dl, mean±SD**	48.68±7.26	47.04±8.00	50.60±11.26	48.72±8.84	0.113
**Platelets(×1000µ/L), mean±SD**	253.26±35	251.78±32	245.32±29	250.12±32	0.422
**WBC(×1000µ/L), mean±SD**	8.23±1.3	7.97±1.1	8.10±1.2	8.10±1. 2	0.899

p-value<0.05 was considered significant. FBS: fasting blood sugar, BUN: blood urea nitrogen, LDL- C : low-density lipoprotein cholesterol, HDL-C: high-density lipoprotein cholesterol, HDL-C: High-density lipoprotein cholesterol ,WBC: White blood cell

No significant relation between sex and platelet aggregation was observed (p=0.886). Pearson correlation test did not show any significant correlation between age and platelet aggregation (p= 0.067, r = 0 .061).

Table 2 shows the data related to basal platelet aggregation and also platelet aggregation after taking aspirin 80 mg/day and different doses of garlic tablets. Aspirin (80mg/day) significantly inhibited platelet aggregation via blocking AA,as compared to the baseline (14.6%±18.4 vs 79.9%±7.0, p<0.001). Inhibition of AA-induced platelet aggregation after using aspirin was significantly more than the group that used three garlic tablets a day, (p<0 .001).

**Table 2:** Platelet aggregation by four agonists in healthy volunteers consumed aspirin and different doses of garlic tablets. (N = 62

**Agonists**	**Base**	**Aspirin ** **80 mg / day ** **N=62**	**GROUP 1** **1 TABLET / DAY** **N = 19**	**GROUP 2 ** **2 TABLETS / DAY ** **N = 22 **	**GROUP 3** **3TABLETS / DAY ** **N = 21**
**AA (0.5 mg/ml)**	79.9±7.05	14.6±18.4*	94.1±15.9	87.9±7.05	87.9±21.6
**ADP ( 20 μmol/l)**	97.7±12.3		79.9±7.05	97.0±19.0	81.2 ±19.7
**Collagen (0.19 mg/ ml)**	74.7±15		98.1±12.2	98.1±12.2	90.1±12.13
**EPN (20 μmol/l)**	64.1±34.5		52.1±29.5	48.8±35.6	47.4±33.5

AA = arachidonic acid, ADP = adenosine diphosphate, EPN= Epinephrine

* p<0.05 was compared between baseline platelet aggregation and Platelet inhibition by aspirin

Based on Table 2, no significant differences were seen in platelet aggregation among three groups that consumed different doses of garlic tablet (one, two or three tablets/day) and no significant differences were observed in comparison with the baseline.

Although platelet aggregation was not changed significantly following consumption of garlic tablets, some cases reported adverse effects after using garlic tablets including nine women (30%) who suffered from heavy menstrual bleeding, the same as those observed with aspirin and also five volunteers who received three garlic tablets daily reported epistaxis.

## Discussion

In patients with acute coronary syndrome who underwent percutaneous coronary intervention (PCI), the 2009 ACC/ AHA guidelines recommend initiating dual antiplatelet therapy with aspirin (162-325 mg daily) and a thienopyridine, either clopidogrel or prasugrel (Kushner et al., 2009 ▶). Studies have reported aspirin and clopidogrel-resistance in some patients justifying the ineffectiveness of these drugs in the prevention of major cardiovascular events (Kuliczkowski et al., 2009 ▶, Angiolillo et al., 2009 ▶, Farré et al., 2010 ▶). So, finding an alternative agent can solve the problem of anti-platelet drugs resistance. The primary aim of this RCT was to introduce an appropriate alternative agent for aspirin. 

The current study was different from the previous ones. One is the increased number of participants in this study compared to others. Another is the use of formulated garlic products available in the market rather than using aged garlic extract (AGE); also, most studies were done *in vitro* rather than a clinical study (Lawson et al., 1992 ▶, Steiner and Li, 2001 ▶, Wagner et al., 1987 ▶). 

Garlic is known as an anti-platelet substance in the literature; however, we were unable to find the underlying mechanism and effective anti-platelet dose of garlic. In this study, aspirin was used as a gold standard anti-platelet drug. Data on average reduction of platelet aggregation caused by the 4 mentioned agonists showed that garlic tablets considerably reduced ADP and EPN; however, their platelet inhibitory effect was not significant.

Another notable point in our study was the bleeding in the volunteers after the use of garlic tablet, but this clinical finding was not confirmed by laboratory tests; Probably, because it is necessary to use methods that can completely simulate intravascular space to see what happens after garlic is used, or maybe it is due to the use of other agonists in platelet aggregation pathway such as thrombin. Although some studies still suggest that the gold standard test for evaluation of platelet aggregation is LTA, this method has some limitations as follow: it requires skilled technician and large sample volume and there is a delay in obtaining results due to assay length; also, this method cannot show what is happening in the vessel and cannot show the interaction between drug and endothelium layer (Angiolillo et al., 2009 ▶).

Additionally, because LTA is subjected to several variables such as concentration of agonist and the LTA value (maximal or late platelet aggregation), the reproducibility of this method is poor (Angiolillo et al., 2009 ▶).

Hence, for determination of anti-platelet effects of garlic, it may be better that the point of care assays including platelet function analyzer-100 (PFA-100) which measures the time taken for anticoagulated whole blood to form a clot in the presence of collagen with either ADP or EPN is used, or Verify –NowTM assays which measure platelet aggregation of whole blood in response to the appropriate agonist (Angiolillo et al., 2009 ▶).

Biochemical studies on the anti-platelet effects of garlic have revealed the effects of garlic on inhibiting platelet aggregation induced by substances such as ADP, collagen, AA, and EPN. In one study on 34 volunteers who received 2.4 g/day fresh garlic for 2 weeks, garlic components could block the effect of collagen, ADP and EPN on platelet aggregation. In this study, increasing the amount of garlic intake, made no significant difference in platelet aggregation (Steiner and Li, 2001 ▶).

As mentioned above, in our study garlic had a more marked influence on the ADP and EPN than the other agonists. These results were proven in *in vitro* and *in vivo* studies (Scharbert et al., 2007 ▶, Hiyasat et al., 2009 ▶, Steiner and Li, 2001 ▶). According to a previous a randomized, double-blind study, garlic blocks the effect of ADP, indicating its antiplatelet effect. In this study, the effect has been noted to be dose-dependent and it was also shown that ajoene blocks the effect of collagen and ADP on platelet aggregation (Steiner and Li, 2001 ▶). In another *in vitro* study, the effect of aqueous extract of garlic on inhibition of platelet aggregation via blocking the effect of ADP, collagen, AA, and EPN was reported to be dose-dependent (Hiyasat et al., 2009 ▶). Also, one paper reviewed the cellular mechanisms involved in garlic’s effect on blocking platelet aggregation induced by ADP, one of the most important of which, suppressed the metabolism of calcium in platelets (Bordia et al., 1998 ▶). As expected, generalizing the results of *in vitro* studies to clinical trials and *in vivo* studies is not always possible. So our findings can be different from those of in vitro studies (Johnson et al., 2001 ▶).

In a randomized, double –blind study of normal healthy individuals (n= 34), the effect of AGE was evaluated at doses between 2.4 and 7.2 g /day (Steiner and Li, 2001 ▶). Platelet aggregation and adhesion were measured at 2 week intervals throughout the study. That study reported that threshold concentration for EPN and collagen increased moderately during AGE administration (2.4 g /day) compared with the placebo, but at the highest dose (7.2 g / day), AGE showed a slight increase in the threshold level of ADP-induced aggregation. 

In our study, by increasing the number of garlic tablets taken per day and also increasing the duration of garlic consumption, similar results comparable to other studies can be achieved. Also, unlike previous studies, in our study, instead of AGE, commercial garlic product (garlic tablet, Nature made) was used. According to some studies, factors such as different levels of sulfur content in products, extraction method and also, the final dosage form of the product (tablet or oil), may affect the potency and efficiency of the final products (Makheja and Bailey, 1990 ▶). Rational relation between the sulfur content of the products and the antiplatelet effect of various commercial garlic products has been reported (Lawson et al., 1992 ▶).

In this RCT, we were unable to determine the effective anti-platelet dose of garlic tablet that could be equal to that of aspirin anti-platelet activity, as assessed by LTA method. So, with these results it is not possible to introduce garlic tablet as an appropriate alternative for aspirin as a cardio-protective agent. Further research is needed to clarify the optimal anti-platelet dose of garlic.

## Note

*Please cite this paper as: *

Shafiekhani m, Faridi P, Kojuri J, Namazi S. Comparison of antiplatelet activity of garlic tablets with cardio-protective dose of aspirin in healthy volunteers:a randomized clinical trial. Avicenna J Phytomed, 2016; 6 (5): 550-557.

## References

[B1] Afzal M, Ali M, Thomson M, Armstrong D (2000). Garlic and its medicinal potential. Inflammopharmacology.

[B2] Agarwal KC (1996). Therapeutic actions of garlic constituents. Med Res Rev.

[B3] Angiolillo DJ, Suryadevara S, Capranzano P, Zenni MZ, Guzman LA, Bass TA (2009). Antiplatelet drug response variability and the role of platelet function testing: a practical guide for interventional cardiologists. Cathet Cardiovasc Interv.

[B4] Angiolillo DJ (2009). Variability in responsiveness to oral antiplatelet therapy. Am J Cardiol.

[B5] Apitz-Castro R, Escalante J, Vargas R, Jain Mk (1986). Ajoene, the antiplatelet principle of garlic, synergistically potentiates the antiaggregatory action of prostacyclin, forskolin, indomethacin and dypiridamole on human platelets. Thromb Res.

[B6] Bordia A, Verma S, Srivastava K (1998). Effect of garlic (Allium sativum) on blood lipids, blood sugar, fibrinogen and fibrinolytic activity in patients with coronary artery disease. Prostaglandins Leukot Essent Fatty Acids.

[B7] Ernst E (1986). Cardiovascular effects of garlic (Allium sativum): a review. Pharmatherapeutica.

[B8] Farré AJL, Tamargo J, Mateos-cáceres PJ, Azcona L, Macaya C (2010). Old and new molecular mechanisms associated with platelet resistance to antithrombotics. Pharmaceut Res.

[B9] Gupta N, Porter TD (2001). Garlic and garlic-derived compounds inhibit human squalene monooxygenase. J Nutr.

[B10] Hiyasat B, Sabha D, Grötzinger K, Kempfert J, Rauwald JW, Mohr FW, Dhein S (2009). Antiplatelet activity of Allium ursinum and Allium sativum. Pharmacology.

[B11] Jain AK, Vargas R, Gotzkowsky S, Mcmahon FG (1993). Can garlic reduce levels of serum lipids? A controlled clinical study. Am J Med.

[B12] Johnson J, Decker S, Zaharevitz D, Rubinstein L, Venditti J, Schepartz S, Kalyandrug S, Christian M, Arbuck S, Hollingshead M (2001). Relationships between drug activity in NCI preclinical in vitro and in vivo models and early clinical trials. Br J Canc.

[B13] Kuliczkowski W, Witkowski A, Polonski L, Watala C, Filipiak K, Budaj A, Golanski J, Sitkiewicz D, Pregowski J, Gorski J (2009). Interindividual variability in the response to oral antiplatelet drugs: a position paper of the Working Group on antiplatelet drugs resistance appointed by the Section of Cardiovascular Interventions of the Polish Cardiac Society, endorsed by the Working Group on Thrombosis of the European Society of Cardiology. Eur Heart J.

[B14] Kushner FG, Hand M, Smith SC, King SB, Anderson JL, Antman EM, Bailey SR, Bates ER, Blankenship JC, Casey DE (2009). focused updates: ACC/AHA guidelines for the management of patients with ST-elevation myocardial infarction (updating the 2004 guideline and 2007 focused update) and ACC/AHA/SCAI guidelines on percutaneous coronary intervention (updating the 2005 guideline and 2007 focused update): a report of the American College of Cardiology Foundation/American Heart Association Task Force on Practice Guidelines. J Am Coll Cardiol.

[B15] Lau BH, Adetumbi MA, Sanchez A (1983). Allium sativum (garlic) and atherosclerosis: a review. Nutr Res.

[B16] Lawson LD, Ransom DK, Hughes BG (1992). Inhibition of whole blood platelet-aggregation by compounds in garlic clove extracts and commercial garlic products. Thromb Res.

[B17] Longo D, Fauci A, Kasper D, Hauser S (2011). Harrison's Principles of Internal Medicine.

[B18] Makheja A, Bailey J (1990). Antiplatelet constituents of garlic and onion. Agents actions.

[B19] Schaffer EM, Liu JZ, Green J, Dangler CA, Milner JA (1996). Garlic and associated allyl sulfur components inhibit N-methyl-N-nitrosourea induced rat mammary carcinogenesis. Canc Lett.

[B20] Scharbert G, Kalb ML (,Duris M, Marschalek C, Kozek-Langenecker SA). 2007. Garlic at dietary doses does not impair platelet function. Anesth Analg.

[B21] Sendl A (1995). Allium sativum and Allium ursinum: Part 1 Chemistry, analysis, history, botany. Phytomedicine.

[B22] Srivastava K (1993). Antiplatelet principles from a food spice clove (Syzgiumaromaticum L). Prostaglandins Leukot Essent Fatty Acids.

[B23] Steiner M, Li W (2001). Aged garlic extract, a modulator of cardiovascular risk factors: a dose-finding study on the effects of AGE on platelet functions. J Nutr.

[B24] Wagner H, Wierer M, Fessler B (1987). Effects of garlic constituents on arachidonate metabolism. Planta Med.

